# The ‘Gopher Sign’: A Clinical Sign to Determine the Adequate Depth of CO_2_ Laser Ablation in Rhinophyma

**DOI:** 10.29252/wjps.8.1.120

**Published:** 2019-01

**Authors:** Muhammad U Javed, Maxwell Morison

**Affiliations:** Welsh Centre for Burns and Plastic Surgery, Morriston Hospital, Swansea, SA6 6NL, United Kingdom

**Keywords:** Gopher sign, CO2 laser, Rhinophyma, Sign

**DEAR EDITOR**

The CO_2_ lasers are widely used for laser ablation of rhinophyma. For the treatment to be successful, it is essential to control the depth of the epidermal vaporization with limited damage to papillary dermis.^1^ Poor recognition of adequate depth and inappropriate selection of energy delivered with CO_2_ lasers can lead to hypertrophic scarring and delayed wound healing.^2^ In this article we describe the new ‘Gopher Sign’; a clinical sign to assist an operator in determining the adequate depth of CO_2_ laser treatment of rhinophyma. 

A case of 80 year old gentleman with moderate rhinophyma is presented and the ‘Gopher Sign’ demonstrated. The patient underwent treatment with CO_2_ laser (Lumenis, Santa Clara, California) aided by use of the plume evacuator. The main bulk of the rhinophyma was ablated using a 2 mm ‘True Spot’ hand piece on Continuous Wave settings; which started at 12 Watts. A layer by layer technique was used and the energy was progressively lowered to maintain the control and minimize potential of over reduction and scarring. After two or three passes visible expression of the contents of the dilated glands is observed as illustrated in Figure 1. 

**Fig. 1:**
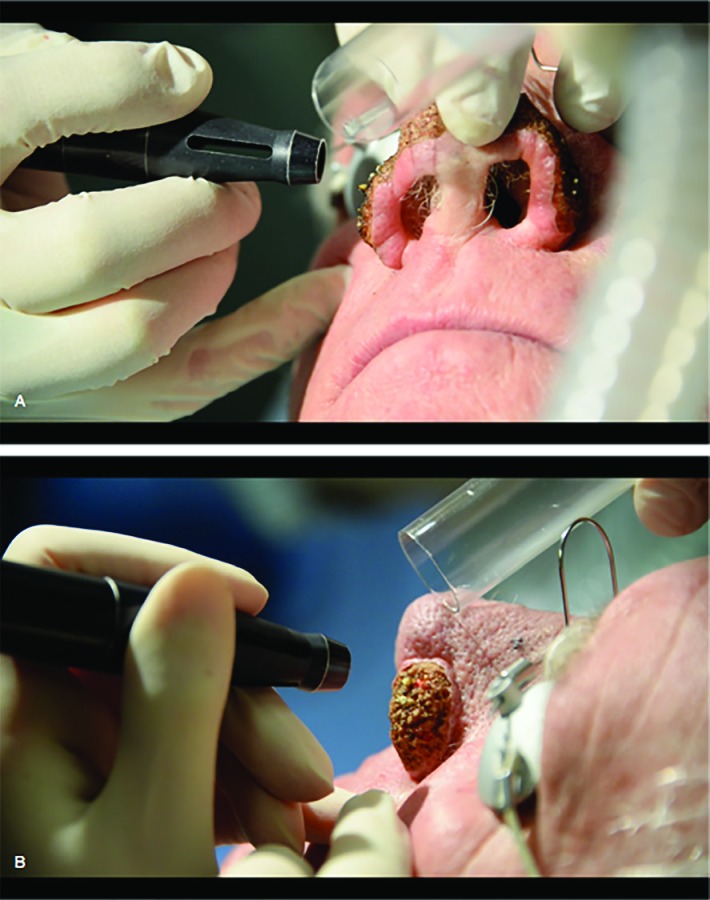
A and B illustrate the ‘Gopher Sign’; expression of glandular content observed in right and left ala of nose

This expression of glandular content, analogous to a ‘Gopher’ reaching out of its burrow signifies early sign of adequate ablation of tissues (Figure 2). Once this sign is observed the operator should reduce the energy for one to three passes. As the bulk is reduced, it is important to progressively lower the energy to maintain control and minimize potential of over reduction and scarring. This usually follows a pattern of 8 watts, 6 watts and 3 Watts until satisfactory end point is achieved. This is followed by the feathering phase using the Ultra Pulse mode of the CO2 laser (2 mm hand piece and settings 125-175 J cm^2^, 10-15 Hz). 

**Fig. 2:**
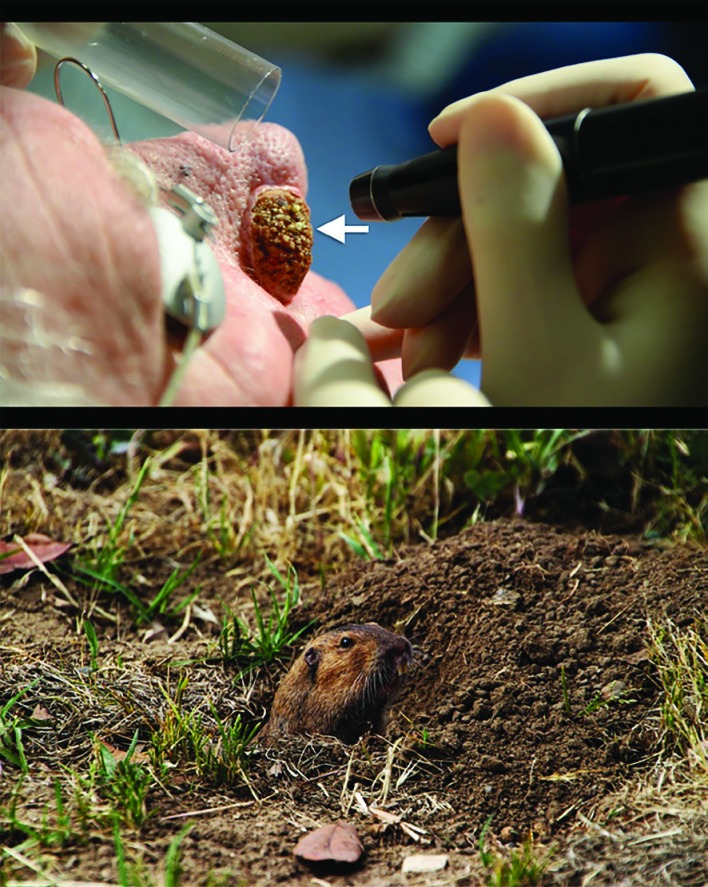
The clinical sign (arrow) is analogous to the Gopher looking out of its burrow

In literature, authors advocate careful selection of energy settings and its application but no author describes the clinical appearance an operator should look for to determine the adequacy of ablation. The rhinophyma is often asymmetrical with variable thickness of tissue and it is very easy to over treat the areas. Gopher sign is a new and simple clinical sign which an operator can use as a guide to early sign of adequate ablation. Hence, as soon as expression of glandular content is noted the operator should consider tapering the energy levels as recommended above. In our experience the Gopher sign is easily identified and is reproducible. We have used this sign reliably in the treatment of over twenty rhinophyma patients and find the ‘Gopher Sign’ to be a very useful adjunct to prevent over treatment. Despite its simplicity, we do recommend caution in its use especially by operators with limited clinical experience in the laser treatment of rhinophymas.

## CONFLICT OF INTEREST

The authors declare no conflict of interest.
